# PET imaging of animal models with depressive-like phenotypes

**DOI:** 10.1007/s00259-022-06073-4

**Published:** 2023-01-16

**Authors:** Daniel Aaron Vazquez-Matias, Erik F. J. de Vries, Rudi A. J. O. Dierckx, Janine Doorduin

**Affiliations:** Department of Nuclear Medicine and Molecular Imaging, University Medical Centre Groningen, University of Groningen, Groningen, the Netherlands

**Keywords:** Major depressive disorder, Depressive-like behaviour, Preclinical imaging, Neuroreceptor, PET, Animal models

## Abstract

Major depressive disorder is a growing and poorly understood pathology. Due to technical and ethical limitations, a significant proportion of the research on depressive disorders cannot be performed on patients, but needs to be investigated in animal paradigms. Over the years, animal studies have provided new insight in the mechanisms underlying depression. Several of these studies have used PET imaging for the non-invasive and longitudinal investigation of the brain physiology. This review summarises the findings of preclinical PET imaging in different experimental paradigms of depression and compares these findings with observations from human studies. Preclinical PET studies in animal models of depression can be divided into three main different approaches: (a) investigation of glucose metabolism as a biomarker for regional and network involvement, (b) evaluation of the availability of different neuroreceptor populations associated with depressive phenotypes, and (c) monitoring of the inflammatory response in phenotypes of depression. This review also assesses the relevance of the use of PET imaging techniques in animal paradigms for the understanding of specific aspects of the depressive-like phenotypes, in particular whether it might contribute to achieve a more detailed characterisation of the clinical depressive phenotypes for the development of new therapies for depression.

## Introduction

Major depressive disorder (MDD) is one of the most prevalent psychiatric disorders worldwide [1]. It presents a clinical course characterised by a broad and heterogenous constellation of symptoms, which may lead to harmful consequences, such as prolonged periods of disability, or even suicidal behaviour. According to data from the World Health Organisation (WHO), around 300 million people worldwide are affected by depression and it is predicted that this disorder will become the first cause of incapacity in terms of disability-adjusted life years (DALYs) by 2030 [1]. This lack of understanding entails a major challenge to both clinicians and fundamental researchers, and may hamper the selection of effective therapeutic schemes, and slow down the development of new therapies. One of the most notorious examples of these knowledge-gaps is the lack of reliable diagnostic auxiliary tools to stage severity and monitor the clinical course of MDD in the managing protocols [2].

Currently, there are no reliable diagnostic biomarkers available to stage patients with MDD or predict the outcome of treatment [3]. Present MDD management protocols are largely based on antidepressant drugs. The use of these antidepressants began as a serendipitous finding after these drugs were prescribed to patients with different illnesses, and improvements in the affective state were found as a side effect [4, 5]. MMD standard treatment includes drugs like selective and non-selective serotonin and norepinephrine reuptake inhibitors, which are used as first line of treatment. However, about 50–70% of MDD patients experience incomplete remission of symptoms after receiving standard antidepressant treatment [6–8]. In view of the high rates of treatment failure with standard antidepressant therapy, and the limited understanding of the pathophysiology, it is necessary to fill the knowledge gaps regarding MDD in order to develop and prescribe more effective treatments. New insights could be obtained from clinical studies, but these studies are expensive, time-consuming and for ethical reasons may only be possible for certain types of research questions. Hence, preclinical research with animal models of depression could help to overcome these hurdles.

In the last decades, an increasing number of research models for depression have been developed in an effort to gain insight in the pathophysiology of MDD and to evaluate the effectiveness of antidepressant therapies [9]. Models to study MDD aim at mimicking different aspects of the biology of depressive phenotypes. These models can vary in complexity from simple interventions such as pharmacological treatments, disruption of the HPA axis, and specific gene knock-outs to sophisticated social interactions in living animals and the response to environmental insults. By manipulating one or more of these variables, researchers induce ‘depression-like behaviour’ in experimental animals as a paradigm of the human depression [10]. Nevertheless, the translatability of the findings in these models to the clinical practice is frequently questioned, because so far, animal models of depression can only replicate some symptoms of MDD, and none of them fulfils the three external criteria (predictive, face and construct validity) for animal models [11–13]. In fact, animal models are not able to replicate the most complex features of MDD like suicidal ideation, or explain phenomena like the time lag between some pharmacological interventions and the onset of their clinical effect. Yet, preclinical research using animal models of depression could provide useful insight in the biological processes that are related to certain MDD symptoms, and with the incorporation of non-invasive molecular imaging techniques like PET, the translation of the preclinical findings to the clinic could be facilitated. PET imaging is a cutting-edge tool suitable for investigation of neuropsychiatric disorders. Thanks to the expanding armamentarium of radiopharmaceuticals, PET can provide information about a wide span of physiological and disease-related processes, such as brain metabolism, neuroreceptors availability and inflammatory responses, among others. However, PET has not yet found a place in the regular clinical assessment of MDD, as it has in other pathologies like Alzheimer’s disease [14, 15].

This review first provides an overview of the animal models used for specific symptoms of depression and then summarises the findings obtained with PET imaging in these animal paradigms of depressive symptoms. To place the results into context, we also compared the findings from PET studies in animal models with the equivalent work done in MDD patients.

## Animal models of depression

Modelling human behaviour in animals is one of the most challenging tasks in neuroscience. Ideally, a phenotype with a high risk to display depressive-like behaviours (e.g. ‘negative coping’ or ‘reduced sucrose preference’) is induced by exposing the animals to similar conditions as the ones believed to trigger specific symptoms in humans [16]. Currently, several paradigms are used to elicit depressive-like phenotypes in animals. Stress has been generally acknowledged as a strong risk factor to induce depressive states. A large number of the current experimental models to study MDD use stress as a factor to trigger depressive-like behaviour in analogy to eliciting depression in humans. In a comprehensive review, Gold proposed that symptoms of depressive disorders can result from an unbalanced adaptive response to ensure survival, in which a hypothalamus–pituitary–adrenal (HPA) axis dysfunction is the main effector. HPA axis response involves multi-level metabolic changes from local brain transmission pathways to peripheral endocrine reactions [17]. Stress can be enforced in experimental animals with different paradigms, such as the despair models based on forced swimming (FS) or learned helplessness (LH), and the chronic stress models that apply repeated social defeat (RSD), chronic (unpredictable) mild stress (CMS/CUMS) and chronic restraint stress (CRS). These paradigms, among others, have proven successful in triggering responses similar to human depression symptoms in different animal species [18, 19]. Depressive phenotypes can also be elicited by other experimental approaches like the use of selective in-bred strains of animals like Flinster-sensitive rats or Wistar-Kyoto rats [9, 20–22]. Pathological models of other brain diseases or systemic illnesses that are accompanied by depressive symptoms in humans, such as epilepsy, hormonal deficiencies, Parkinson’s disease and systemic infection, have also been used [23–25]. Depressive-like behaviour in animals was initially only assessed with measurable outcome parameters that were based on various depressive symptoms, such as anhedonia, helplessness and anxiety, with the purpose of screening for activity of potential antidepressant drugs. An overview of depressive-like behaviours displayed by different animal models is presented in Table 1.

**Table 1:** Summary of the findings from behavioural tests in different depressive paradigms

	Experimental paradigm	Anhedonia	Sleep disturbances	Despair behaviour	Anxiety	Social interaction	Cognitive impairment
Despair	Forced swimming			Increased immobility [26–29]			
	Foot shock			Increased escape failure [30, 31]			
Chronic stress	Repeated social defeat	Reduced sucrose preference [26, 27, 32] Increased reward intracranial self-stimulation thresholds [33]	Sleep impairment and circadian disruptions [34]	Increased immobility [27]	Decreased motility and exploratory activity [26, 27, 32] No differences in motility and exploratory activity [35]		Higher swimming escape latency [27] No changes in object recognition [32] Decreased rearing [27]
	Chronic restraint stress	Reduced sucrose preference [36, 37]		Increased immobility [36, 37]	Decreased motility [28]		Decreased object recognition [37]
	Chronic unpredictable mild stress	Reduced sucrose preference [38–40]		Increased immobility [40]	No differences in motility [38, 40] Decreased motility [39]		
In-bred	Flinders sensitive rats	Increased sucrose preference in females [21]		Increased immobility [21]	Decreased motility and exploratory activity [21]		Decreased object recognition Higher swimming escape latency [21]
Depressive-like behaviour secondary to other pathologies	Hypothyroidism	Reduced sucrose preference [23]			No differences in motility [23]		
	Status epilepticus	Reduced sucrose preference [24]		Increased immobility [24]			
	Striatal denervation (Parkinson model)			Negative correlation between Immobility and 5HT receptor binding [25]			
	Submitted social status					Negative correlation between depressive behaviour and 5HT binding [41]	
	Peripheral inflammation (Interferon administration)	Reduced sucrose preference [42]					
	Neuropathic pain (nerve ligation)			Increased immobility [43]	No differences in motility [43]		

## PET imaging in animal models of depression

Not only behavioural outcome parameters are important for our understanding of depression phenotypes. Nowadays, several physiological processes, including brain metabolism, neurotransmission, and inflammatory responses have been identified to be involved in depression. These processes potentially represent targets for investigation of depressive phenotypes using imaging techniques like PET.

### Imaging of brain metabolism with [^18^F]FDG-PET

2-deoxy-2-[^18^F]fluoro-D-glucose ([^18^F]FDG)-PET can be used to measure cerebral glucose metabolism, which is usually interpreted as ‘brain activity’. In [^18^F]FDG-PET studies, the [^18^F]FDG signal increases when glucose demanding processes are induced, like an increase of synaptic activity or an inflammatory response [44, 45]. The [^18^F]FDG signal decreases when brain activity is reduced, like in case of synaptic dysfunction, neural degeneration, or reduced physical activity [46]. The most common approaches to analyse [^18^F]FDG-PET studies are quantification of the cerebral metabolic rate (CMRglu) and the regional quantification of the tracer uptake a using standardised uptake value (SUV) or a ratio of SUVs among regions (SUVr), either by the analysis of predefined regions of interest, or by a voxel-based analysis. [^18^F]FDG-PET has been applied in several studies in animal models of depression to investigate the regional metabolic responses to different depressive-like phenotypes (Table 2). The following sections summarise the [^18^F]FDG-PET imaging findings in different types of preclinical depression models.

**Table 2:** Summary of the findings of [^18^F]FDG-PET of the brain in different depressive paradigms

Brain area		Despair		Chronic stress			In-bred	Depressive-like behaviour secondary to other pathologies	
		Forced swimming	Foot shock	Chronic unpredictable mild stress	Repeated social defeat	Chronic restraint stress	Flindes sensitive rats	Hypothyroidism	Status epilepticus
Cortex	Piriform	Decreased [29]		Decreased [38]	Decreased [32]				
	Orbital	Decreased [29]			Decreased [32]	Decreased [36]		Decreased [23]	Decreased [24]
	Insular	Decreased [29]			Decreased [32]				Decreased [24]
	Entorhinal	Decreased [48]			Increased^#^, decreased^#^ [32]	Decreased [36]	Decreased [21]		Decreased [24]
	Motor	Increased [29]			Decreased [32]			Decreased [23]	
	Sensory	Increased [48]		Decreased [39]	Decreased [32]				
	Retrosplenial			Decreased [39]					
	Auditory cortex			Increased [38]					
				Decreased [39]					
	Cingulate				Decreased [32]	Decreased [36]		Decreased [23]	Decreased [24]
	Medial prefrontal				Decreased [32]	Decreased [36]			Decreased [24]
Subcortical nuclei	Hippocampus	Decreased [29, 48]	Decreased [30]		Decreased [32]	Decreased [36]			Decreased [24]
	Amygdala	Decreased [48]		Decreased [60]	Decreased [32]	Decreased [36]			Decreased [24]
	Septal nuclei		Increased [31]	Decreased [38]					
	Thalamus			Decreased* [39]	Decreased [32]				Decreased [24]
	Globus pallidus			Decreased [39]					
	Habenular nuclei		Increased [31]						
	Striatum	Increased [29]		Decreased [60]	Decreased [32]	Decreased [36]		Decreased [23]	
	Hypothalamus				Increased^#^, decreased^ [32]				
	Accumbens					Decreased [36]		Decreased [23]	
	Olfatory bulbs					Decreased [36]	Decreased [21]		
Midbrain	Inferior colliculus	Decreased [29, 48]		Decreased [38, 39]	Increased^#^, decreased^ [32]		Decreased [21]		
	Periaqueductal grey			Decreased [38]	Increased^#^, decreased^ [32]		Decreased [21]		
	Ventral tegmental area			No changes [40]	Increased^#^, decreased^ [32]		Decreased [21]		Decreased [24]
	Cerebellum	Increased [29, 48]		Increased [60]					Decreased [24]

*Postero-medial thalamic nuclei. ^These areas were reported as brainstem. ^#^Different timepoints

#### Despair paradigms

The despair paradigms are among of the most frequently used models to induce depressive-like behaviour in preclinical research. In fact, several studies have used [^18^F]FDG-PET to evaluate brain glucose metabolism in the helpless depressive-like phenotype triggered by the despair models. One of the first studies by Jang and co-workers evaluated the [^18^F]FDG-PET response in a despair model. They used forced swimming to induce helplessness in male Sprague–Dawley rats. This study found decreased glucose metabolism (voxel-based analysis) in the orbital and insular cortices, hippocampus and inferior colliculi, while an increased tracer uptake was observed in the cerebellum and striatum [29]. These findings partly agree with the meta-analysis of Fitzgerald et al. [47], who also found ‘inactivation’ of the insular cortex, and regions in temporal and frontal cortices in depressed patients, but observed ‘activation’ of the hippocampus in patients. Jang and co-workers also performed a study using [^18^F]FDG-PET to explore the effect of the antidepressant fluoxetine on forced swimming induced helplessness [48]. In this study, they found that forced swimming caused a decrease in glucose metabolism (voxel-based analysis) in the insular cortex, hippocampus, amygdala and inferior colliculi, and an increase in cerebellum and motor sensory cortices in both the fluoxetine-treated group and the control group. Forced swimming exposed animals that did not receive the antidepressant displayed lower [^18^F]FDG uptake in the entorhinal and insular cortices than forced swimming exposed animals that were treated with the antidepressant [48]. A meta-analysis revealed that antidepressant medication influences the activity of several brain circuits in patients, including regions such as the amygdala, anterior cingulate, prefrontal and insular cortices, putamen and hypothalamus. Results from both animal and human studies therefore indicate a decrease in the activity of the insular cortex as a result of the depressive paradigm and ‘activation’ of this brain region after antidepressant therapy [49]. Both animal studies displayed similar [^18^F]FDG uptake patterns after the exposure to forced swimming and [^18^F]FDG uptake patterns resemble the activation/deactivation patterns seen in patients in meta-analyses [47, 49].

In contrast to the findings from forced swimming induced helpless animals, learned helplessness induced by foot-shock exposure — another well-established despair-based paradigm — resulted in more limited changes in [^18^F]FDG uptake. For example, the study by Seo et al. found that male Sprague–Dawley rats exposed to foot-shock with a ‘negative coping’ phenotype displayed lower glucose metabolism (voxel-based analysis) in the left ventral dentate gyrus and the adjacent hippocampal regions than animals with a ‘positive coping’ phenotype [30]. This finding might be related to the hippocampal atrophy that has been reported in depressive patients [50, 51]. A distinctive characteristic of the dentate gyrus is the presence of active neurogenesis in adult life. In this context, the reduction in [^18^F]FDG uptake in the ‘negative coping’ animals could be related to a decrease in cell proliferation in the dentate gyrus, which has also been reported in an [^18^F]FLT-PET study [52], and studies in other animal models and in depressed patients [53, 54]. However, the implication of the dentate gyrus cell density on the development of depressive-like behaviour remains controversial, as studies have shown poor correlation between the onset of depressive-like behaviours and the cell proliferation in this structure [55, 56]. Another study in male Sprague–Dawley rats found that glucose metabolism (voxel-based analysis) in the habenular and lateral septal nuclei positively correlated with some behavioural parameters associated with the coping style towards learned helplessness, such as the number of times the animals pressed the lever to avoid the noxious stimuli and the time used to finish the test. In addition, [^18^F]FDG uptake in these regions correlated positively with the tracer uptake in other cortical areas and subcortical nuclei, but correlated negatively with uptake in the dorsal part of the hippocampus [31]. This study presents a compelling approach, as it is focusing not only on the effect of the paradigm on brain activity but also on the correlation of brain activity in sensitive areas with that in other areas of brain. The habenular nuclei participate in the mesolimbic reward pathway and it has been found that silencing of the circuit between the lateral habenular nuclei and the ventral pallidum reduced immobility (despair behaviour) in depressive-like phenotypes [57]. Similarly, a reduction in activity in the septal nuclei, measured as c-fos expression, has been reported, in particular in negative coping animals [58].

Both the forced swimming and learned helplessness paradigms triggered despair-like behaviour in animals, which is usually quantified as the time to escape or actively avoid noxious stimuli [59]. Yet, the effect of forced swimming induced helplessness on brain metabolism activity measured with [^18^F]FDG-PET differs from the effect of foot-shock induced learned helplessness. Forced swimming induced a widespread response in some cortical areas and the hippocampus, while the response to the foot-shock learned helplessness focused mainly on the hippocampus. These differences could be interpreted as the forced swimming paradigm being capable of eliciting a stronger effect on the metabolic brain activity than the foot-shock paradigm. However, this could also be an echo of the differences in complexity between the two paradigms.

#### Chronic-stress paradigms

An alternative approach frequently used to generate a depressive-like phenotype is the use of chronic-stress paradigms such as CUMS, RSD and CRS. Similar to the despair models, the stress models not only revealed heterogeneous effects on [^18^F]FDG uptake between the different models but also between studies using the same model. For example, Hu et al. described that male Sprague–Dawley rats that underwent a CUMS protocol showed a reduction in glucose metabolism (voxel-based analysis) in the piriform cortex, septal nuclei, left colliculus and periaqueductal grey matter; and an increase in [^18^F]FDG uptake in the left auditory cortex [38]. On the other hand, Zhou et al. reported that exposure to CUMS caused a decrease in [^18^F]FDG uptake (SUVr) in the nucleus of the inferior colliculus, the retro-splenial agranular area, secondary sensory and primary auditory cortices, thalamic postero-medial nucleus and globus pallidus [39]. Both studies display similar CUMS-induced metabolic deactivation patterns, yet not identical. The pattern in Zhou’s study described a wide spread deactivation, whereas in Hu’s study metabolic deactivation seemed to be more limited, even showing activation in some areas described as deactivated in Zhou’s study. The study by Baptista and co-workers investigated [^18^F]FDG uptake (SUV) in the ventral tegmental area (VTA) in male Wistar rats that underwent the CUMS paradigm, but could not find any differences between CUMS exposed and the control animals. This study also assessed the effect of the treatment with ketamine, which did not affect the [^18^F]FDG uptake in the VTA either [40]. Laeken and co-workers compared [^18^F]FDG uptake (brain normalised uptake values) after CUMS exposure with tracer uptake after sub-chronic administration of corticosterone (as positive stress control). This study showed that the CUMS protocol displays only partially the brain inactivation/activation pattern that is induced by corticosterone administration [60]. Taken together, these studies indicate that the CUMS model has proven capable not only to induce depressive-like behaviour but also to modify the brain activity measured with [^18^F]FDG-PET. In most studies, CUMS induces a deactivation of brain activity, but the effects of CUMS on [^18^F]FDG uptake show a large variability in distribution pattern and magnitude among the studies. This could be explained by subtle differences in the stress protocol, as for example the timing and the order in which the stressors are applied is not standardised to make the model ‘unpredictable’. Different stressors require responses from different brain areas and thus might contribute to the variability found in these studies.

Using the repeated social defeat (RSD) paradigm as chronic stress model, Kopschina-Feltes et al. found a reduction in [^18^F]FDG uptake (SUV) in several brain areas in male Wistar-Unilever rats that underwent the RSD protocol for 5 consecutive days [32]. One day after completion of the RSD protocol, the animals displayed a reduction in [^18^F]FDG uptake in motor, sensory, cingulate and entorhinal cortices. Twenty days after RSD, the animals showed a progressive reduction in brain metabolism in the aforementioned brain areas, but also reduction in [^18^F]FDG uptake in the medial prefrontal, orbitofrontal, insular and piriform cortices and in subcortical structures like hippocampus, striatum, thalamus, hypothalamus, amygdala, cerebellum and brainstem. However, these changes in [^18^F]FDG uptake were transient, as tracer uptake had normalised again 3 and 6 months after exposure to RSD. These results imply that some brain areas are more sensitive to the RSD protocol than others. The initial hypometabolism in these sensitive areas could subsequently have triggered a similar reaction in other areas [32].

[^18^F]FDG-PET has also been used to evaluate the metabolic activity in the brain of animals subjected to the chronic restraint stress paradigm. Wei’s and co-workers found a reduction in [^18^F]FDG uptake (SUVr) in several brain regions of restrained male Wistar rats, such as the entorhinal, left medial prefrontal, cingulate and insular cortices, antero-dorsal hippocampus, right nucleus accumbens, caudate-putamen, amygdala and olfactory bulbs. The reduction in brain glucose metabolism in the nucleus accumbens, hippocampus, caudate-putamen, auditory, entorhinal and medial prefrontal cortices of restrained animals correlated with a decrease in sucrose preference, a measure of anhedonia. Likewise, increased immobility in forced swimming test correlated well with the change in [^18^F]FDG uptake in nucleus accumbens, amygdala, caudate putamen, hippocampus, auditory, cingulate, insular, entorhinal and medial prefrontal cortices [36].

Overall, the studies on the metabolic activity in the chronic-stress models described in this review displayed a heterogenous decrease in [^18^F]FDG uptake that seem to involve more areas than observed in the studies on despair models. These [^18^F]FDG uptake reductions were found in both cortical and subcortical areas, suggesting the deactivation of a broader spectrum of sensitive regions in the chronically induced depressive-like phenotypes.

#### In-bred depressive phenotypes

Another common approach in preclinical research on depressive disorders is the use of in-bred strains prone to develop depressive-like behaviour, like the Flinders-sensitive strain and the Wistar-Kyoto rat strain. Thiele et al. investigated the cerebral glucose metabolism (voxel-based analysis) in Flinders-sensitive male and female rats after simple handling and behavioural tests at young (2 months) and older (7 months) age. The Flinders-sensitive animals in this study displayed more immobility in the forced swimming test and memory impairment compared to the Sprague–Dawley control animals, but did not display anhedonia-like symptoms (using the sucrose preference test). This study showed hypometabolism in the temporal lobe, entorhinal cortex, brainstem and olfactory bulbs of the Flinders-sensitive animals at both ages when compared to control animals [21].

#### Depressive-like behaviour secondary to other pathological phenotypes

Depressive symptoms are not exclusively featured in psychiatric disorders. These symptoms frequently partake in the clinical manifestations of diverse syndromes. For this reason, the study of depressive symptoms and depressive-like behaviour is also relevant for other pathological conditions and the corresponding animal models. For example, the hormone deficiency models frequently display depressive traits as part of the triggered symptoms. The study of Yu et al. evaluated depressive-like behaviour and [^18^F]FDG uptake (SUVr) in male Wistar rats after a regime of propylthiouracil to induce pharmacological hypothyroidism. This study found a reduction in [^18^F]FDG uptake in the striatum frontal, cingulate and motor cortices of hypothyroid animals, which was accompanied by a progressive decrease in sucrose preference [23]. A similar approach was used in the study by Khayum et al., who evaluated the interaction between oestradiol replacement therapy and exposure to CMS. This study did not reveal any significant main effect of the CMS intervention on [^18^F]FDG uptake (SUV) in any brain region. However, it found a significant effect of oestrogen depletion on the development of despair behaviour in the FS test. Moreover, reduced [^18^F]FDG uptake was found in the frontal cortex and thalamus of stressed oestrogen-depleted rats, when compared to stressed animals that received oestrogen replacement therapy [61].

Another example is the pilocarpine-induced model of status epilepticus. In the study of Zanirati et al., a decrease in [^18^F]FDG uptake (SUVr) in cingulate, entorhinal, insular, medial prefrontal and orbitofrontal cortices, hippocampus, thalamus, amygdala, cerebellum and ventral tegmental area was observed 29 days after the status epilepticus induction. The follow-up evaluation 46 days after status epilepticus induction revealed persisting low [^18^F]FDG uptake in the same areas, except for the cerebellum. This study also found a correlation between the reduction in sucrose preference and the reduction in [^18^F]FDG uptake in the whole brain and in brain regions, such as the amygdala, thalamus, entorhinal cortex, insular and prefrontal cortices. In addition, correlations between the immobility in FS test and the [^18^F]FDG uptake in hippocampus, amygdala and cingulate cortex were found. Furthermore, the assessment of the brain network in this study found similar decreased in brain connectivity in both epileptic ‘depressed’ and ‘non-depressed’ phenotypes concluding that the network alterations found in these animals could be better explained by the epileptic phenotype, and not by the depressive-like one [24].

#### Concluding remarks on [^18^F]FDG PET in animal models in depression

[^18^F]FDG-PET has been applied in different models of depression to study the brain response in an attempt to characterise the depressive-like phenotypes used in preclinical research. [^18^F]FDG-PET studies consulted for this review displayed a broad range of results, with different patterns of increased and decreased [^18^F]FDG uptake, suggesting that activation or deactivation of different brain regions in response to the experimental stimuli could highly depend on the specific experimental paradigm employed. The general tendency observed in the preclinical [^18^F]FDG-PET studies indicates that regional glucose consumption in animal models of depression is decreased in areas like the entorhinal, orbital and cingulate cortices and subcortical structures associated with the limbic system, such as hippocampus, amygdala, thalamus and striatum [21, 23, 24, 29, 30, 32, 36, 39, 48, 60]. In addition, some studies displayed an increase in metabolic activity in regions like the cerebellum and habenular nuclei [29, 31, 48, 60]. Preclinical [^18^F]FDG-PET findings, such as the deactivation of regions like the frontal, cingulate insular and temporal cortices, are in agreement with the results of a meta-analysis on [^18^F]FDG-PET in depressed patients [62]. However, since there is not a clear gold-standard to diagnose depression, the deviating results in animals cannot be discarded as part of depressive phenotypes yet. In preclinical studies, contradicting results were observed in some brain regions between different animal models. For example, the septal nuclei showed increased [^18^F]FDG uptake in the chronic stress paradigm, but decreased tracer uptake after foot shock-induced helplessness [31, 38]. Furthermore, some regions displayed time-dependent effects, with an initially decrease in [^18^F]FDG uptake shortly after exposure to RSD, followed by an increase in tracer uptake at later timepoints [32]. Overall, the results suggest that cortical regions display changes (usually a reduction) in glucose metabolism more often than subcortical nuclei. Changes in [^18^F]FDG uptake seem to be more evident in chronic stress models [32, 36, 38, 39, 60], than in the despair paradigms and models [29–31, 48] with depressive-like behaviour related other pathology [23, 24]. This could be explained by the fact that exposure to the stressor in the chronic stress models lasts longer than in the other paradigms. Overall, the interpretation [^18^F]FDG uptake in the brain at a regional level remains difficult. Despite that the general assumption is that [^18^F]FDG uptake is directly related to neuronal activity, a regional change in glucose demand does not necessarily imply the direct involvement of that specific brain area in induction of depressive symptoms. [^18^F]FDG PET might therefore not yield clear answers about the causal relationships and mechanisms that trigger depressive-like behaviour. However, it could provide a direction for subsequent research. Like animal studies, [^18^F]FDG-PET studies in humans show discrepant results for the glucose consumption in patients with MDD, when compared to healthy controls [62, 63]. This implies that regional [^18^F]FDG-PET is probably not a suitable tool to identify a specific signature for depressive phenotypes [62, 63]. One of the main challenges for [^18^F]FDG-PET data analysis in depressive-like phenotypes is the complexity of the signals observed. Due to the complexity observed in [^18^F]FDG-PET data, it is becoming increasingly clear that there is a need for more advanced analysis of brain activity. Dimensionality reduction methods, such as independent component analysis, principal component analysis, factor analysis, scale subprofile modelling, graph methods, among others, have been demonstrated as suitable methodologies to assess [^18^F]FDG-PET data in a multivariate approach, and describe the connectivity in the brain instead of independent regional effects [64].

The assessment of brain connectivity in depressive phenotypes has emerged as a compelling approach to gain a better understanding of the depressed brain. The brain connectivity has been widely addressed using magnetic resonance imaging methods (fMRI, diffusion tensor imaging, etc.) [65–67]. For example, the fMRI study of Gass et al. in congenitally learned helplessness susceptible animals — inbreed Sprague–Dawley rats — found local increased internodal role and reduced local efficiency in default mode network hubs such as an in the anterior cingulate and prelimbic cortices of the susceptible animals in comparison with the non-susceptible controls [68]. [^18^F]FDG-PET data can also be used to describe and measure the brain networks in human and animal depressive phenotypes [69, 70]. Su et al. [^18^F]FDG-PET study in depressed patients found impaired connectivity in network hubs such as pars triangular, prefrontal and visual cortices, as well as, in the salience and language networks of MDD patients in comparison with healthy volunteers [71]. The finding of impairment in the salience network could be related to the reduction of the metabolic activity in the insular cortex seen in the regional studies in human and animal phenotypes. Both of these connectivity studies suggest different brain network changes in their respective depressive phenotypes. Yet, neither the findings of the preclinical fMRI study nor the ones of the clinical [^18^F]FDG-PET have not been compared to [^18^F]FDG-PET data of other depressive-like phenotypes or larger human datasets. Therefore, [^18^F]FDG-PET brain connectivity studies in animal models for depression remain as an open question for future research.

### Neuroreceptor imaging

PET makes it possible to target specific receptor populations in the brain and presents an opportunity to improve our understanding of neuropsychiatric disorders, such as MDD. PET imaging of neuroreceptors is a valuable tool to investigate the monoaminergic theory of MDD [72]. This theory suggests that an imbalance of monoaminergic neurotransmitters, such as serotonin (5HT), dopamine and norepinephrine, is responsible for the development of depressive symptoms [4]. The vast majority of the MDD treatments is targeting one or more monoamine neurotransmitter systems. PET imaging of several neurotransmitter systems has already been applied in a variety of animal models of depression (Table 3).

**Table 3:** Summary of the main findings from PET imaging studies on neuroreceptors in animal models of depressive-like phenotypes

Neurotransmission system	Target	Tracer	Experimental model	Affected brain regions	Findings	Reference
Serotonin	5HT_1A_ receptor	[^18^F]MeFWAY	Striatal dopaminergic denervation with 6-OHDA	Hippocampus	Increase in 5HT_1A_ receptor availability (BP_ND_) in dopamine denervated animals	[25]
		[^18^F]MeFWAY	Forced swimming	Hippocampus	Increase in 5HT_1A_ receptor availability (BP_ND_) after forced swimming exposure	[77]
		[^18^F]MPPF	Dominance-submission role in social status paradigm	Raphe nuclei, amygdala, hippocampus and anterior cingulate cortex	Reduction in 5HT_1A_ receptor availability (BP_ND_) in submitted animals	[41]
	5HT_2A_ receptor	[^11^C]MDL100907	Repeated social defeat (2 days)	Olfactory bulb, frontal cortex, amygdala, hippocampus, pons and medulla	No effects (BP_ND_)	[35]
	Serotonin transporter	[^11^C]DASB	Corticosterone administration	Cortex, hippocampus, striatum and thalamus	Decreased serotonin transporter availability (BP_ND_)	[89]
		[^18^F]ADAM	NMDA withdrawal	Frontal cortex, striatum, thalamus, hypothalamus and midbrain	Decreased serotonin transporter availability (SUR)	[91]
Dopamine	D_2_ receptor	[^11^C]raclopride	Inflammatory cytokine administration	Caudate and putamen	Decrease of D_2_ receptor availability (BP_ND_) after interferon-α exposure	[42]
	Dopamine transporter	[^18^F]FECNT	Inflammatory cytokine administration	Caudate and putamen	No effects (BP_ND_)	[42]
Norepinephrine	α_2_ receptor	[^11^C]yohimbine	Comparison of sensitive to standard experimental animals	Frontal cortex, striatum, thalamus, pons and cerebellum	Decreased α_2_ receptor availability (V_T_) in depression sensitive animals	[109]
Glutamate	mGlu5 receptor	[^11^C]ABP688	Spinal nerve ligation (neuropathic pain)	Prefrontal, somatosensory and retrosplenial cortices	Increased availability (BP_ND_) of mGlu5 receptors	[43]
		[^11^C]ABP688	Spinal nerve ligation (neuropathic pain)	Insular and piriform cortices, accumbens and endopiriform nuclei	Decreased availability (BP_ND_) of mGlu5 receptors	[43]
Opiate	κ, δ and μ opioid receptors	[^18^F]FDPN	Spinal nerve ligation (neuropathic pain)	motor and insular cortices and caudate-putamen	Decreased opioid receptor availability (BP_ND_)	[116]

#### The serotonergic system

The serotoninergic receptor system consists of at least 14 receptor subtypes and is one of the most studied neurotransmission systems in relation to depression [73]. Only 2 of these 5HT receptor subtypes have been investigated with PET in animal models of depression so far: 5HT_1A_ and 5HT_2A_. The 5HT_1A_ receptor is one of the most abundant serotonin receptors in the brain, and it has been associated with multiple functions such as serotonin dependant transmission regulation and synaptogenesis [74, 75]. 5HT_1A_ receptor density in both rodent and human brains is the highest in regions such as the hippocampus, hypothalamus and frontal, cingulate and entorhinal cortices [76]. PET imaging has made it possible to evaluate these receptors in models of different depressive-like phenotypes using ligands such as *N*-{2-[4-(2-methoxyphenyl)piperazinyl]ethyl}-*N*-(2-pyridyl)-*N*-(*trans*-4-[^18^F]fluoromethylcyclohexane) ([^18^F]MeFWAY) and 2′-methoxyphenyl-(*N*-2′-pyridinyl)-*p*-[^18^F]fluoro-benzamidoethylpiperazine ([^18^F]MPPF). [^18^F]MeFWAY-PET displayed a reduction in 5HT_1A_ receptor availability expressed as the non-displaceable binding potential (BP_ND_) in the hippocampus of female Sprague–Dawley rats after exposure to forced swimming [77]. Similarly, [^18^F]MeFWAY-PET also revealed a reduction in the hippocampal 5HT_1A_ receptor availability (BP_ND_) in 6-hydroxydopamine denervated male Sprague–Dawley rats, which correlated with immobility in the forced swimming test [25]. Another study using the ligand 4-[^18^F]fluoranyl-N-[2-[4-(2-methoxyphenyl)piperazin-1-yl]ethyl]-N-pyridin-2-ylbenzamide ([^18^F]MPPF) in female cynomolgus monkeys characterised as depressed or not depressed by behavioural observation displayed a reduced 5HT_1A_ receptor availability (BP_ND_) in anterior cingulate cortex, hippocampus, amygdala and raphe nuclei of socially submitted monkeys (second-ranking animals) [41]. Thus, the studies available for this review highlight a common and strong reduction of the 5HT_1A_ receptor availability in the hippocampus across the different depressive-like phenotypes. However, it should be noted that two of these studies only assessed the hippocampus in their PET imaging design and the potential response of other brain regions to the paradigms was not explored. A metanalysis of 10 studies with 218 depressed patients and 261 healthy controls showed similar results as those found in monkeys, indicating a reduced 5HT_1A_ receptor binding (BP_ND_) in hippocampus, raphe nuclei, insular and anterior cingulate cortex in depressed patients [78].

All PET studies on the 5-HT_1A_ receptor in animal models of depression and depressive patients used an antagonist as the tracer so far. In contrast, autoradiography studies with the 5-HT_1A_ agonist 8-OH-DPAT have been performed that provide complementary information [79, 80]. Naudon and coworkers used autoradiography to show that [^3^H]-8-OH-DPAT binding in prefrontal, cingulate, motor and sensorial cortices, hippocampus, dentate gyrus, amygdala and raphe nuclei of helpless mice was increased relative to control mice [73]. Treatment with fluoxetine reduced the binding of the agonist again in cingulate cortex and raphe nuclei. Since agonists only bind to the high-affinity state of the receptor, these findings suggest that the density of receptors in the high-affinity state is increased in depressive phenotypes, whereas PET studies show that the total 5-HT_1A_ receptor density is decreased. Amigo et al. used autoradiography to study the functionality of 5-HT_1A_ and demonstrated that 8-OH-DPAT-induced [^35^S]GTPγS binding (as a proxy for 5-HT_1A_ functionality) in the dorsal raphe nucleus was reduced after chronic exposure to cortisone. Treatment with fluoxetine further decreased receptor functionality [74].

The 5HT_2A_ receptor has been investigated using the PET ligand (*R*)-[1-[2-(4-fluorophenyl)ethyl]piperidin-4-yl]-(2-methoxy-3-[^11^C]methoxyphenyl)methanol ([^11^C]MDL100907) in male Wistar-Unilever rats submitted to the RSD protocol. However, no significant differences in tracer uptake between control and RSD defeated animals were observed in any brain region [35], which may be ascribed to the short duration of the RSD protocol. In contrast, increased 5HT_2A_ receptor availability (BP_ND_) has been reported in animals exposed to the CUMS paradigm [81], which is in agreement with the analysis of post-mortem samples of patients with history of MDD [82]. The 5HT_2A_ receptor density in hippocampus, amygdala, prefrontal cortex, striatum and olfactory structures [83] in both humans and animals has been associated with the response to therapy with selective serotonin reuptake inhibitors [84].

Serotonin levels can be regulated by modulation of the synthesis of the neurotransmitter. 5-Hydroxytryptophane (5-HTP) is a precursor in the biosynthesis of serotonin and has been labelled with ^11^C for PET imaging. Although 5-[^11^C]HTP has been successfully used in humans and non-human primates, the tracer proved unsuitable for quantification of serotonin synthesis in rodents and therefore has not been used in animals of depression [85]. Serotonin synthesis, however, has been studied using autoradiography with α-[^14^C]methyl-L-tryptophan (α-[^14^C]MTrp). This study by Kanemaru and co-workers addressed the activation of tryptophan hydroxylase in Flinders Sensitive and Resistant rats. This study showed that tryptophan hydroxylase activation inhibitor did not induce any differences in the serotonin synthesis in the FSL animals, but did so in the FRL, which suggests that tryptophan synthesis is more activated in the depressive-like phenotype [86].

The serotonin transporter (SERT) is one of the main effectors in the regulation of serotonin metabolism in the brain, and it has been frequently related to the development of depressive phenotypes in both animals and humans [87, 88]. The SERT is encoded by the Slc6a4 gene and has been studied as a relevant molecular target to assess depressive phenotypes, using the ligand [^11^C]3-amino-4-(2-dimethylaminomethylphenylsulfanyl)- benzonitrile ([^11^C]DASB). A [^11^C]DASB-PET study was performed in male and female C57BL/6J Slc6a4^+/+^ mice that underwent chronic corticosterone administration as a chronic stress model. Corticosterone-treated animals displayed lower [^11^C]DASB binding (BP_ND_) in the cortex, hippocampus, striatum and thalamus, which was confirmed post-mortem by quantification of SERT levels with western-blot. This study also evaluated the SERT availability in Slc6a4^+/−^ and Slc6a4^−/−^ mice in comparison with the wild type Slc6a4^+/+^ mice, and found a decreased SERT availability not only in Slc6a4^−/−^ mice but also in heterozygote Slc6a4^+/−^ mice [89]. Based on the decrease in [^11^C]DASB binding in heterozygote animals, it was suggested that the SERT availability can be affected by polymorphism of the receptor, but this was not explored in a post-stress condition. Yet, Slc6a4^+/−^ animals have been suggested as a potential model to study the effects of SERT polymorphisms in the serotonin-transporter-linked promoter region of the Slc6a4 gene [90]. A combined study [^11^C]DASB-PET with ex vivo biodistribution in male CB1-KO and wild-type mice subjected to CRS displayed a lower SERT availability (SUVr using the cerebellum as a reference region) in the cortical brain areas in CB1-KO animals under basal conditions and in wild-type mice subjected to chronic stress in comparison to wild-type controls under basal condition [37]. These results suggest a modulatory role of CB1 in stress response. Shih et al. studied SERT availability in a depressive phenotype induced by withdrawal of N-methyl-D-aspartic acid (NMDA) in male Sprague–Dawley rats. They found a decrease in the specific uptake ratio (SUR) of N,N-dimethyl-2-(2-amino-4-[^18^F]fluorophenylthio)benzylamine ([^18^F]ADAM) in frontal cortex, striatum, thalamus, hypothalamus and midbrain of animals after NMDA withdrawal compared to their control counterparts. In addition, this study found an increase in immobility time in the FS test as a sign of depressive-like behaviour [91]. Thus, the available PET studies display a trend towards a reduction of the SERT availability in cortical and subcortical regions like pre-frontal cortex, striatum and thalamus. These results show little similarity with the findings in 5HT_1A_ and 5HT_2A_ receptors. This could be explained by the fact that the SERT studies did not used the typical paradigms to induce depressive-like behaviours, which were used in the assessment of the 5HT_1A_ and 5HT_2A_ receptors. The experimental approaches used in the SERT studies, however, assessed the occurrence of depressive-like behaviours by measuring sucrose preference or immobility in the FS test after the interventions. On the other hand, the preclinical SERT study is in agreement with a metanalysis of 18 studies with 364 MDD patients and 372 healthy subjects that found significant reductions SERT availability in subcortical regions like amygdala, midbrain, striatum, thalamus and brainstem of depressed patients, and no changes in SERT in cortical areas [92].

#### The dopaminergic system

The dopaminergic system is closely involved in diverse brain functions, such as the reward circuit and motor execution among others. Five different subtypes of dopamine receptors are known so far: D_1_, D_2_, D_3_, D_4_ and D_5_ [93]. According to the monoaminergic hypothesis, unbalanced dopaminergic transmission has been proposed to play a role in depressive disorders. The role of dopaminergic transmission in depressive-like phenotypes has been explored by imaging the availability of key dopaminergic receptors, the dopamine transporter (DAT) and dopamine synthesis. Despite the vast number of publications on PET imaging of the dopaminergic system in depressed patients, only a few papers have been published on dopamine imaging in animal models of depression. These preclinical studies focus on PET imaging of the dopaminergic D_2_/D_3_ receptors and the DAT.

Dopamine D_2_ and D_3_ receptors have been identified as relevant players in the development of depressive-like behaviours and the response to antidepressant drugs [94, 95]. Felger and co-workers performed a PET study on male and female rhesus monkeys that were treated with interferon-α as a model of peripheral inflammation in a cross-over design. When treated with interferon-α, these monkeys showed a decreased sucrose preference, and a reduced striatal D_2_/D_3_ receptor availability (BP_ND_) determined with 3,5-dichloro-*N*-[[(2*S*)-1-ethylpyrrolidin-2-yl]methyl]-2-hydroxy-6-[^11^C]methoxybenzamide([^11^C]raclopride)-PET. Subsequently, [^11^C]raclopride-PET scans were performed in combination with an amphetamine challenge in order to evaluate the effect on dopamine release. This experiment displayed significantly more displacement of the tracer in the putamen of the interferon-α treated animals in comparison with the control treatment [42]. These available PET imaging studies assessing the dopaminergic receptors did not show any overexpression of D_2_ receptors in the prefrontal cortex and hippocampus, as was previously found in depressive paradigms like FS and RSD [96, 97]. A [^11^C]raclopride-PET study in humans by Peciña and co-workers found that patients with depression (*N* = 26) showed higher D_2/3_ receptor availability (BP_ND_) in striatal regions than controls (*N* = 16) [98]. This suggests that the preclinical paradigms used to study the depressive phenotype in monkeys are not suitable to represent the characteristics of the dopaminergic system in the human phenotype.

In contrast to the dopamine receptor studies, the ligand 2β-carbomethoxy-3β-(4-chlorophenyl)-8-(2-[^18^F]fluoroethyl)nortropane ([^18^F]FECNT) with PET did not display any differences in DAT availability between interferon-α treated monkeys and their control counterparts for any brain region [42]. Similar to this study in monkeys, a meta-analysis on DAT imaging that included 12 studies with 209 depressed patients and 314 healthy controls revealed no altered DAT availability in patients with MDD as compared with healthy controls [99].

Other targets in the dopaminergic system have been investigated using autoradiography. For example, a study by Novick and co-workers, using the D_1_ receptor ligand [^3^H]-SCH23390, found decreased D1 receptor density in the striatum of the stress sensitive Wistar-Kyoto rats, when compared to normal Wistar rats [100]. In contrast, D_1_ receptor density was higher in Wistar-Kyoto rats than in regular Wistar rats.

#### The glutamatergic system

The glutamatergic system includes 26 known receptors and is responsible for a significant proportion of the excitatory signals in the nervous system [101, 102]. It is intimately related to other transmission systems, such as the dopaminergic and serotoninergic system. Consequently, glutamatergic receptors could be relevant targets for the study of depressive disorders. One of the best characterised glutamatergic receptors is the mGluR5 receptor. This receptor has been subject to investigation in the spinal nerve ligation model for depressive-like behaviour in response to neuropathic pain. PET imaging with the mGluR5 ligand (*E*)-*N*-[^11^C]methoxy-3-[2-(6-methylpyridin-2-yl)ethynyl]cyclohex-2-en-1-imine ([^11^C]ABP688) revealed an increase in the availability (BP_ND_) of mGluR5 in the medial prefrontal, somatosensory and retrosplenial cortices, the caudal part of the prelimbic area and the medial septum. It also showed a decrease in the receptor availability in the insular and piriform cortices, olfactory tubercle and sectors of corpus callosum and nucleus accumbens [43]. The results described in the literature regarding the physiological behaviour of mgluR5 remain ambiguous and do not entirely align with the findings of the [^11^C]ABP688-PET study. Some findings like the increase in mGluR5 density in the nucleus accumbens and hippocampus are corroborated by post-mortem studies using depressive paradigms, such as social isolation and learned helplessness [103–105], while other findings of the PET study like the increase in mGluR5 availability in the medial prefrontal cortex were not observed in post-mortem studies in rats that underwent RSD [106]. On the other hand, [^11^C]ABP688-PET in patients (*N* = 11) with depression also revealed lower mGluR5 binding (BP_ND_) in the hippocampus, thalamus, prefrontal, cingulate and insular cortices, as compared to healthy volunteers (*N* = 11). This study also found that the severity of depression was negatively correlated with mGluR5 binding in the hippocampus [107]. Post-mortem and in vivo imaging studies have found and an increase in glutamate levels in the prefrontal cortex of MDD patients [108], which could explain the reduced availability of mgluR5 in imaging studies as a results of the high concentrations of glutamate in depressive phenotypes.

So far, other glutamatergic receptors have not been investigated in animal models of depression with PET yet, but the relevance of the glutamatergic system for the general functioning of the brain makes glutamatergic imaging an area with lots of potential for research in the future.

#### The norepinephrinergic system

Norepinephrinergic neurotransmission has been hypothesised to be related with the development and progression of depressive disorders. The α_2_ receptor is one of the receptors in this system with a well-documented role in depressive phenotypes. However, its behaviour in patients and animal models remains controversial. A study by Landau et al. assessed the α_2_ receptor distribution in female Flinders sensitive line (FSL) rats, Flinders resistant line (FRL) rats and Sprague–Dawley rats using the ligand [^11^C]methyl(1*S*,15*R*,18*S*,19*R*,20*S*)-18-hydroxy-1,3,11,12,14,15,16,17,18,19,20,21-dodecahydroyohimban-19-carboxylate ([^11^C]yohimbine) with PET. [^11^C]yohimbine-PET showed a decrease in α_2_ receptor binding (V_T_) in frontal cortex, striatum, thalamus, pons and cerebellum of FSL animals when compared to their FRL and Sprague–Dawley counterparts [109]. Depressive paradigms, such as CUMS, were shown to increase the expression of α_2_ receptors in the hypothalamus [110], which seems to contradict the finding of Landau’s [^11^C]yohimbine-PET study. The results of this PET study are also not in line with findings from studies in patients, which displayed increased expression of α_2_ receptor in hypothalamus, locus caeruleus, hippocampus and pre-fontal cortex [111, 112]. These results suggest a limited construct validity of the FSL model for the norepinephrinergic neurotransmission-related aspects of MDD.

#### The opioid system

The expression of κ and δ opioid receptors is reduced in depressive-like phenotypes, and these receptors have been widely proposed to mediate the responses that elicit depressive-like behaviour in animals [113–115]. A study by Thompson et al. explored the availability of opioid receptors in a male Sprague–Dawley rats with a depressive phenotype induced by neuropathic pain, using PET with 6-O-(2-[^18^F]fluoroethyl)-6-O-desmethyldiprenorphine ([^18^F]FDPN) as the ligand. This study found decreased opioid receptor availability (BP_ND_) in multiple brain regions like motor and insular cortices, caudate-putamen, contralateral posterior insula and contralateral M1/M2 [116]. These results seem to agree with the existing literature that indicates that knocking out or pharmacological blocking of κ and δ receptors in mice leads to the development of depressive phenotypes [117]. However, [^18^F]FDPN binds to the three known opioid receptor families, and therefore, it does not provide any insight into the specific receptor involved. On the other hand, PET studies with specific tracers aiming to characterise the role of opioid receptors in depression have displayed a reduction of the μ-receptor in the pre-frontal cortex of depressed patients [118]. PET studies investigating the κ-receptor, however, did not show any differences in between MDD patients and healthy volunteers [119].

### PET imaging of glial activation and the blood–brain barrier

#### Glial activation

Disbalance of the local and peripheral immune responses has been increasingly acknowledged as part of the depressive phenotypes, both in humans and animal models [120]. Peripheral cytokines, as well as the recruitment of inflammatory cells have been widely associated with the depressive symptoms, supporting the inflammatory hypothesis of depression [121]. Among the physiological processes related to inflammatory responses, glial activation has often been used as imaging target to study neuroinflammation. The most frequently used molecular target to study the activation of glial cells is the 18kD translocator protein (TSPO), formerly known as the peripheral benzodiazepine receptor. Various tracers have been developed for PET imaging of this target [122]. Some PET studies have demonstrated the induction of an inflammatory response in animal models of depressive-like phenotypes. For example, the study by Wang et al. described an increase in the tracer uptake measured as the percentage of the injected dose (%ID/g) of the TSPO ligand N,N-diethyl-2-(2-(4-(2-[^18^F]fluoroethoxy)phenyl)5,7dimethylpyrazolo[1,5a]pyrimidin-3-yl)acetamide ([^18^F]DPA714) in the hippocampus of male Sprague–Dawley rats exposed to CUMS [123]. Similarly, the study of Kopshina-Feltes et al. found an increased uptake of the TSPO ligand N-butan-2-yl-1-(2-chlorophenyl)-N-[^11^C]methylisoquinoline-3-carboxamide ([^11^C]PK11195) in male Wistar-Unilever rats that were exposed to the RSD protocol. In particular, a transient increase in [^11^C]PK11195 uptake (SUV) was observed in the orbital, entorhinal, insular and frontal association cortices 11 days after the RSD protocol, while tracer uptake remained increased only in the frontal association cortex 26 days after RSD [32].

Neuroinflammation assessment with PET in depressive phenotypes secondary to other pathological models also showed that neuroinflammation was associated with depressive-like behaviour. For example, Bertoglio et al. used a model for epilepsy in male Wistar Han rats, which developed depressive-like behaviour as a comorbidity. This study used PET with the TSPO ligand 2-[6-chloro-2-[4-(3-[^18^F]fluoranylpropoxy)phenyl]imidazo[1,2-a]pyridin-3-yl]-N,N-diethylacetamide ([^18^F]PBR111) and demonstrated an increased tracer binding (SUV) in limbic structures, such as the hippocampus, temporal lobe, thalamus and hypothalamus. This activation of microglia correlated well with a reduction in sucrose preference and an increase in immobility in the FS test between 2 and 12 weeks after the induction of status epilepticus [124]. Dobos and colleagues used an intracerebral injection of lipopolysaccharide (LPS) in male C57B1/6J mice as an acute inflammation model of depression and found an increased [^11^C]PK11195 uptake in the whole brain. This study also investigated inhibition of the enzyme indoleamine-2,3-dioxygenase (IDO). IDO activity is commonly enhanced during inflammatory responses. In Dobos’s study, IDO inhibition decreased the immobility time which can be interpreted as a reduction in depressive-like behaviour; however, it did not affect the uptake of the inflammatory marker [125].

Taken together, these preclinical studies all showed that depressive-like behaviour was associated with glial activation, but the affected brain regions were dependent on the model applied. Consequently, the distribution of glial activation in these models does not exactly represent the situation in MDD patients. For example, patients (*N* = 81) with history of 10 years or more of untreated depressive symptoms displayed higher TSPO tracer binding (V_T_) of the ligand N-[[2-(2-[^18^F]fluoranylethoxy)phenyl]methyl]-N-(4-phenoxypyridin-3-yl)acetamide ([^18^F]FEPPA) in prefrontal, anterior cingulate and insular cortices. Furthermore, this TSPO-PET imaging study revealed that clinical parameters of depression, such as illness duration, duration without treatment and duration of antidepressant therapy, are predictors of the binding of the TSPO ligand [126]. A possible explanation for this discrepancy in the distribution of glial activation between animal models and patients could be differences in time frames, as human TSPO imaging studies displayed characteristic tracer uptake patterns after years of untreated illness, while the animal experiments are carried out within days or weeks after the induction of depressive symptoms.

#### Blood–brain barrier

The disruption of function of the blood–brain barrier (BBB) has been proposed and investigated as a relevant contributor in the pathophysiology of psychiatric affections including depressive disorders [127]. The function of the BBB can be assessed with PET by quantifying the binding of 2-(3,4-dimethoxyphenyl)-5-[2-(3,4-dimethoxyphenyl)ethyl-[^11^C]methylamino]-2-propan-2-ylpentanenitrile ([^11^C]verapamil) to the efflux transporter P-glycoprotein (Pgp). In physiological conditions, [^11^C]verapamil is actively cleared from the tissue by the action of the Pgp preventing its accumulation in the tissue. This method was chosen in the study of de Klerk et al. in male Wistar rats exposed to the foot-shock paradigm to induce learned helplessness. They found an increased volume of distribution (V_T_) of [^11^C]verapamil in the brain of the helpless animals in comparison with the controls, which can be interpreted as a reduced clearance of the tracer due to the reduction of the biological function of the Pgp. In the same study, De Klerk and co-workers also addressed the effect of the antidepressant therapy with venlafaxine in foot-shock exposed animals, which showed a reduced in [^11^C]verapamil V_T_ in the venlafaxine treated animals in comparison with the animals treated with vehicle solution. This result suggests that the antidepressant treatment increased the function of the BBB [128]. Additionally, this result also coincides with the findings made in a human study with MDD patients by the same research group, which found lower [^11^C]verapamil accumulation in the brain of patients treated with antidepressants in comparison with the healthy control group, suggesting an increase of the BBB activity due to the administration of antidepressants in both human and animal depressive phenotypes [129].

## Discussion

Depressive disorders, such as MDD, present a substantial societal and economic burden, which is expected to increase in the foreseeable future. Animal models for depression have typically been used to produce behavioural phenotypes suitable to predict the response of depressive symptoms to therapeutic interventions. This review explored the use of the different animal models for depressive phenotypes with the aim to describe imaging phenotypes associated with different mechanistic theories related to depressive disorders. These models included not only the traditional paradigms that mimic symptoms of depression but also alternative pathological models that display secondary depressive symptoms as part of their clinical course. The understanding of depression represents a challenging endeavour due its complexity and the limitations to decompose the processes involved in its onset and progression. Behavioural animal experimentation often faces hard criticism due to a mix of scepticism regarding the representation of the complex human faculties in less complex animals, and a relative low output in the production of effective treatments for the neuropsychiatric illnesses. Despite the continuous debate about the value of animal experimentation in the psychiatric field, modelling depressive-like symptoms in experimental animals remains a relevant approach to identify the biological processes underlying the disease. Confirming the findings from animal models in patients and exploring the differences between human and animal depressive phenotypes are great opportunities for the application of PET imaging.

The interpretation of PET data from both humans and animal phenotypes is not exempt from challenges. These challenges include the limited temporal and spatial resolution, and a wide variability of depressive phenotypes in both laboratory and the clinic. Furthermore, there are also methodological constrains that might affect the translatability of findings from animal models to patients. For example, the use of anaesthetics in animal studies has been already identified as a confounding factor that can hamper the translatability of imaging studies [130–132]. Additionally, differences in the processing and quantification of the images can also debilitate the translatability of PET methods. For instance, partial volume effects are frequently not accounted for in animal studies due to the lack of anatomical CT or MRI data in the PET stand-alone preclinical systems. The choice of quantification methods can also influence the translatability of preclinical PET. Semiquantitative outcomes such as SUV could be affected by non-specific binding and regional blood flow changes, while use of simplified pharmacokinetic methods can introduce bias in the estimation of outcome parameters like the V_T_ and BP_ND_ [133].

Animal paradigms to model depressive symptoms possess limitations, as they encompass the sum of multiple tasks as input for the system. This complex input introduces variability in the brain responses and makes it virtually impossible to distinguish what features of the resulting images are attributable to the executed task in the study. For example, the forced swimming task involves a motor outcome that is mediated by cortical structures which might also be involved in the affective response. In contrast to patient data, in animal studies, it is possible to control the effect of different confounding factors that have large influence in the outcome of human studies, such as age, and treatment history or the time of the progression of the symptoms. One of the main limitations found in this review is the fact that most of the studies performed in both animals and patients are analysed in a ‘region of interest’ approach which corresponds with a modular view of the brain in which functions are dependent on specific brain areas. The main disadvantage of this approach is that the results obtained do not describe the distribution of entire networks across the brain, but only restricts to determined areas of interest previously described in literature that are hypothesised to display changes after the experimental intervention. Further incorporation of data-driven methods to analyse PET data might overcome this limitation and offer a clearer view of the effect of the model interventions on the brain networks in the studied phenotypes.

Despite the above-mentioned limitations, PET imaging in animal models of depressive-like phenotypes has been able to confirm several results related to the effects of depressive phenotypes on brain activity that were found with PET and other imaging modalities in depressed patients. In addition, several findings on neuroreceptor expression in animal models were corroborated by molecular imaging and post-mortem studies on human brains. Given the limited number of PET studies on animal models of depression performed so far, the available studies do not provide enough information to confirm mechanisms in the different depressive phenotypes, but provide a valuable insight that could not be obtained with post-mortem analyses.

This review shows that in a large number of studies, [^18^F]FDG PET was used as the imaging method, despite the fact that [^18^F]FDG is a relatively unspecific tracer and results may be difficult to interpret. The frequent use of [^18^F]FDG so far is likely related to the general availability of this tracer. As PET imaging progresses, new radiopharmaceuticals and methodology to analyse PET data are constantly expanding the capabilities to track relevant processes hypothesised to influence depressive and depressive-like phenotypes. This expansion will hopefully lead to the validation of new PET imaging methods and validated biomarkers for the clinical assessment of patients with depressive symptoms, selection of the most adequate treatment and monitoring of treatment outcomes. Current PET techniques have allowed to investigate relevant processes related to depressive phenotypes, such as brain activity and dopaminergic and serotoninergic receptor availability. Yet, there is still a wide range of open questions regarding the understanding of the depressed brain and depressive-like phenotypes that could be addressed using PET imaging methods. One of these question concerns to the exploration of the imaging of other molecular targets that have been implicated with depressive phenotypes such as the cholinergic, adenosinergic and GABAergic neuroreceptor systems [134, 135]. Similarly, it is necessary to expand the study of the dopaminergic, serotoninergic and glutamatergic neuroreceptors systems including the receptor families that have not been investigated yet, as well as, the use of genetically modified organism models to address potential receptor variations due to genetic polymorphisms. Another question is the gender variability in the modelling of depressive symptoms in preclinical research. Paradoxically, the majority of the preclinical research has been performed in males despite a higher prevalence of depression in women [16]. The studies consulted for this review were no exception. This does not limit to the inclusion of exploring the imaging phenotypes in both genders, but also should include the targeting of the neuroactive steroid receptors which is possible with current PET methods [136, 137]. Another promising question suitable to address with PET methods is the assessment of the disconnection hypotheses. This subject has been explored mostly in humans using mostly [^18^F]FDG-PET and MRI methods; however, with the introduction of synaptic density imaging with PET tracers like [^11^C]UCB-J, it has become possible to quantify the availability of the SV2A protein as biomarker of synaptic connectivity. [^11^C]UCB-J—PET was used by Holmes et al. to evaluate synaptic density regional distribution in depressed patients. This study found reduced SV2A availability in the dorsolateral-prefrontal and cingulate cortices and hippocampus of severely depressed patients compared with the healthy controls, which correlated with the severity of the depressive symptoms [138]. [^11^C]UCB-J -PET data can also serve as an input for connectivity studies to describe brain networks [139], which suggest a compelling alternative approach for the study of brain networks that needs to be explored in both human patients and animal models.

## Conclusion

This review has recapitulated the main behavioural findings in animal models for depression and the characterisation of the imaging phenotypes that they produce with PET imaging methods. PET imaging has demonstrated to be a versatile and valuable resource for the study biological processes hypothesised to be linked to depressive-like behaviour in animals and depressive symptoms in patients. The studies consulted in this review provide a deeper description of the wide and complex mechanisms behind depressive-like phenotypes in different biological systems. Although several of these imaging findings have displayed similarities with the imaging of human depressive phenotypes, they are not entirely corresponding. However, despite the discrepancies, these findings provide a deeper understanding of the modelling of the depressed brain, and this insight will hopefully influence the further development of the therapeutic approaches.

## Data Availability

Not applicable
